# Effect of PGR pulse treatments and ethylene precursor and inhibitors on Scots pine micropropagation

**DOI:** 10.1186/1753-6561-5-S7-P150

**Published:** 2011-09-13

**Authors:** Vadim Lebedev, Konstantin Shestibratov

**Affiliations:** 1 Branch of Shemyakin and Ovchinnikov Institute of Bioorganic Chemistry RAS, Pushchino, Russia

## Background

Conifer clonal forestry as a form of plantation forestry has great potential advantages. However, vegetative propagation of conifers, especially that of mature trees, is quite problematic. These species also are most recalcitrant objects for cultivation in vitro. For this reason the development of an effective system for clonal micropropagation of some conifers is still an important aim. Scots pine (*Pinus sylvestris* L.) is one of the most widespread conifers in the world and in Russia. This important species has high economic value. Among *Pinus* species, Scots pine is especially difficult to deal with in culture [1]. We investigated influence of pulse treatment with BA, GA and auxins on clonal micropropagation of Scots pine. In addition effects of ethylene precursor and inhibitors were analyzed.

## Methods

Seeds were collected in 2008 from open pollinated trees growing in the Republic of Belarus (Gomel Region) and were stored at 4-5 °C until used. Seeds were washed under running tap water for 24 h, surface-sterilized in 15% H_2_O_2_ for 30 min, rinsed in sterile water and germinated on wet filter paper in plastic containers. The germinating seeds were incubated for 10-11 days at 23±1 °C in the dark. Hypocotyls of the seedlings were trimmed to 5-7 mm upper cotyledons, sterilized in 0.2% Hg(NO_3_)_2_ for 4 min, rinsed in sterile water and placed vertically on hormone-free medium PM1 [2], containing 3% sucrose, 0.25% Gelrite gellan gum (Sigma, USA). After one week (BA treatment) or 6-7 weeks (auxin treatment) explants were used for experiments. For shoot induction explants were treated in an aqueous 50 or 100 mg/l BA solution buffered with 0.1 M MES during 0.5, 1.5 or 4 h. For elongation newly formed shoots were treated in 0.2, 1 or 5 mg/l GA3 during 0.5, 1, 2 or 4 h. For rooting pulse treatment by 50 or 100 mg/l NAA and IBA for 6 or 24 h were used. After treatments explants were transplanted on hormone-free medium: MS and PM1 (BA treatment), PM1 (GA treatment), 1/2 DCR (auxin treatment). Effects of various ethylene inhibitors, including AgNO_3_ (10 and 40 μM) and CoCl_2_ (10 and 100 μM), and the ethylene precursor ethephon (3 and 10 mg/l) also were studied in our experiments. Culture conditions were 23±1 °C and a photoperiod of 16 h. Rooted shoots were transferred to a peat-perlite (3:1) mixture and acclimatized in a greenhouse under gradually decreasing humidity conditions during 3-4 weeks.

## Results and conclusions

The best results after BA pulse treatment were obtained at 4 h exposure to 100 mg/l solution (Table 1). Using this time, about 74.2% of explants formed an average of 3.5 buds. The proportion of vitrified explants was higher on MS medium (up to 76%) than on PM1 medium (up to 33%). It is interesting that we observed shoot regeneration on tips of cotyledons.

**Table 1:** Effect of BA pulse treatment on shoot formation in Scots pine.

Treatment, mg/l - hours	Medium	Vitrification, %	Necrosis, %	Explants with shoots, %	Shoots/explant
0-1.5	PM1	33.3	16.7	0.0	-
	
	MS	63.6	50.0	0.0	-

50-0.5	PM1	20.5	12.2	75.6	3.1
	
	MS	75.5	15.5	71.4	3.1

50-1.5	PM1	12.8	2.1	53.2	2.6
	
	MS	73.5	11.0	40.8	2.8

50-4	PM1	5.7	3.6	62.3	3.0
	
	MS	50.0	12.5	69.0	2.4

100-0.5	PM1	27.8	3.6	64.8	3.2
	
	MS	44.6	6.7	60.7	2.9

100-1.5	PM1	6.3	11.1	56.3	2.8
	
	MS	45.2	13.9	54.8	2.6

100-4	PM1	3.2	8.8	74.2	3.5
	
	MS	23.8	12.5	50.0	1.7

The previous studies showed that optimal treatment duration and the concentration of cytokinin are different for each conifer species. For instance, for explants of *P.canariensis* the best exposure time was 4-8 h in 22.5 mg/l BA solution [3]. High concentrations or prolonged exposure can completely suppress shoot formation. Bud induction in *P. wallichiana* was not observed at concentrations above 135 mg/l BA and time more than 3 h [4].

Pulse treatment with 0.2, 1 or 5 mg/l GA solution did not improve shoot elongation. Addition of AgNO_3_ to medium decreased vitrification and death of explants in comparisons with CoCl_2_ and ethephon. The treatment with 50 mg/l NAA during 6 h resulted in 44% rooted shoots after 12 weeks of culture, whereas pulse treatments with IBA resulted in 13-19% root formation (Table 2). An average of 1.5-3.2 roots per shoot were produced after NAA treatment and only single root per shoot after IBA treatment.

**Table 2:** Effect of auxin pulse treatment on root formation in Scots pine.

Auxin	Treatment, mg/l-hours	Rooting, %	Root/shoot	Root length, mm
control	water-24	3.1	1.0	2.0
NAA	50-6	43.8	1.5	16.5
	50-24	34.4	1.8	17.5
	100-6	37.5	3.2	14.9
	100-24	21.9	2.6	15.8
IBA	50-6	15.6	1.0	40.8
	50-24	12.5	1.0	21.5
	100-6	18.8	1.0	14.2
	100-24	12.5	1.0	12.0

**Figure 1:**
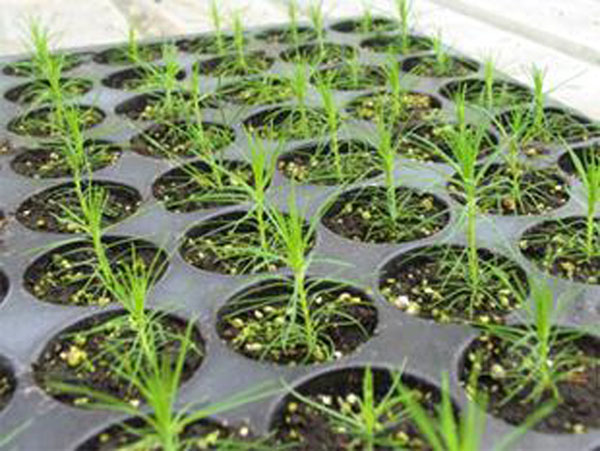
Acclimatized plants of Scots pine in the greenhouse.

Similar results were obtained when auxin pulse treatment was applied on other *Pinus* species. When shoots of *P.ayacahuite* were incubated in 18.6 mg/l NAA for 8 h, up to 40% of the shoots were rooted [5]. An exposure of 6 h to 203 mg/l IBA solution resulted in 27.4% rooted shoots of *P. wallichiana*[*4*]. Rooted plants of Scots pine were successfully acclimatized in the greenhouse with the survival rate 80-90% (Figure 1).
